# The role of occupational therapy in primary care mental health services: a short report

**DOI:** 10.1017/S1463423625100212

**Published:** 2025-06-23

**Authors:** Arabella Hely, Claire Pearce

**Affiliations:** Faculty of Health, University of Canberra, Bruce, ACT, Australia

**Keywords:** mental health, occupational therapy, primary health

## Abstract

This paper is a short report considering the role of occupational therapy in providing mental health services in primary care. Primary care is the first point of contact most people have with the healthcare system. Occupational therapists have a role working with people with mental illness but the role of an occupational therapist working in mental health in a primary care setting is not well understood. Common interventions discussed in the literature included lifestyle approaches, assessment and information gathering, and the teaching of skills for daily living. There was a clear divide in the literature regarding the use of generic or specialist (occupation-focused) roles. Physical health was often prioritized over mental health concerns. Limited research examined both the population group of people with mental health concerns and the practice setting of primary care, identifying the need for further research to articulate the role of occupational therapy in working with mental health in primary care settings.

## Introduction

Mental health is ‘a state of mental well-being that enables people to cope with the stresses of life, realize their abilities, learn well and work well, and contribute to their community’ (World Health Organisation, 2025). A mental illness or mental disorder is a condition that is clinically diagnosable and significantly impacts one’s ability to think, feel, and interact with others (Department of Health and Aged Care, 2012). Whilst there are individual risk factors which contribute to a person’s mental health, the COVID-19 global pandemic highlighted that mental health is significantly impacted by external social factors. The pandemic also highlighted the need to shift away from institutional-based care to community care, with a need for integrated interdisciplinary care to deliver prevention and early intervention and coordinated care for chronic conditions (Murtagh *et al.*, 2021).

*Primary healthcare* is an inclusive, cost-effective strategy that aims to organize and improve national health systems so that health and well-being services are easily accessible to all communities (Bolt *et al.*, 2019a; World Health Organization, 2024). It follows a non-diagnostic, holistic view of a person and their care, which aligns with the values of occupational therapy (Muir, 2012). *Primary care* is a subset of primary healthcare. It serves as the first point of contact an individual has with a health system (The World Health Organization, 2024). Primary care encompasses a wide range of public, private, and non-government services. Primary care providers include general practitioners, nurses, and allied health providers (Australian Institute of Health and Welfare, 2025), including occupational therapy. This report considers the setting of primary care rather than the broader concept of primary healthcare.

Primary care is the ideal practice context for using an integrated, non-reductionist and multidisciplinary approach to a person’s health. This holistic view of a person can be highly effective in therapy (Ee *et al.*, 2020). Occupational therapists work with people and communities to enhance their ability to engage in their desired occupation, that is the things they want to do, need to do or are expected to do, which give their life meaning and purpose (WFOT, 2012). Two recent reviews considered the role of occupational therapy in primary care (Bolt *et al.*, 2019b; Donnelly *et al.*, 2023). Both these reviews concluded that there is a role for occupational therapy in primary care, but further work is needed to define and promote the role, with potential to shift from an individual intervention focus to a preventive, community-based approach. Neither of the reviews drew specific conclusions regarding the occupational therapy’s role in primary care in supporting people with their mental health.

Occupational therapists work with people with mental health challenges to support full participation in life. Occupational therapists may also use their skills to work in generic roles such as case managers and group facilitators (Sammells *et al.*, 2022). When assuming these roles, occupational therapists retain their occupational focus, prioritizing their client’s daily activities and emphasizing practical aspects of role performance and well-being while helping them overcome practical barriers and to develop skills (Occupational Therapy Australia, 2023). Occupational therapy’s unique contribution to mental health services lies in recognizing the complex interplay between client factors, activity demands, and environmental contexts and in examining the relationship between physical and mental health (Burson *et al.*, 2017). Occupational therapy has historically been challenging to define in general mental health settings (Wilding, 2009; Parker, 2001) and in specific settings such as primary care.

## Methods

The broad research question for this report was: *What is the role of occupational therapy in providing mental health services in primary care*? To provide a structured search strategy, the authors followed Arksey and O’Malley’s scoping study framework (2005) excluding the final optional step of consultation which has been deferred until the research identified through this review is conducted. Due to the limited literature available, Levac *et al.*’s (2010) recommended approach of an iterative approach to identifying studies and extracting data was followed, resulting in a narrative review of the included literature.

**Figure 1.**
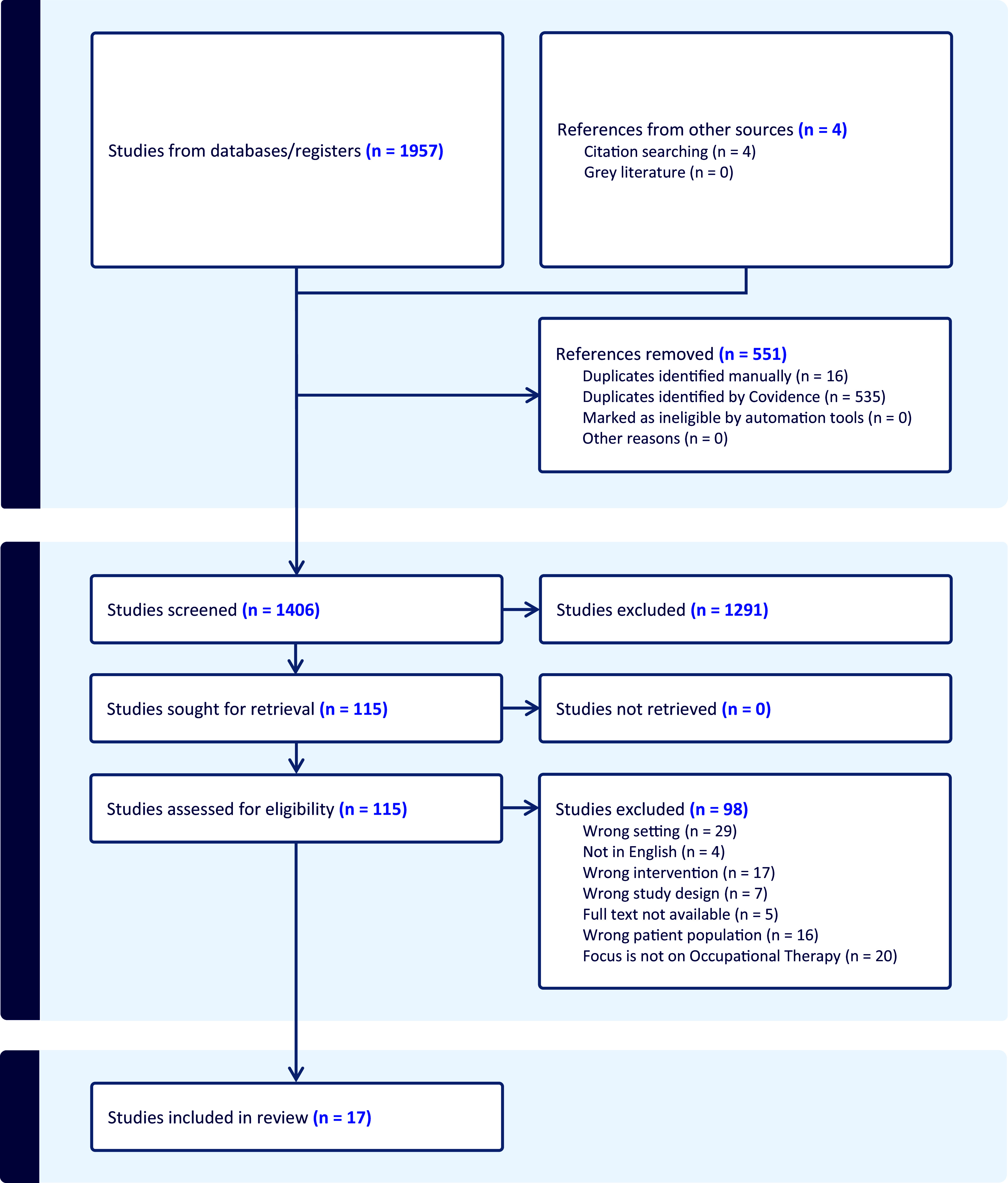
PRISMA 2024 flowchart of articles identified and screened in Covidence (n.d).

Online databases CINAHL Plus, Medline, Academic Search Ultimate, APA PsycInfo, and Web of Science were used to gather relevant literature. Articles not published in English and abstracts for which no full text was available were excluded. No limitations were placed on an article’s publication date or country of origin. Search terms used in this literature review are as follows: ((‘occupation* therap*’ OR ‘occupation* science’) AND (mental OR psychological OR psychiatric OR anxiety OR depression OR psychosis) AND (‘primary health care’ OR ‘primary health care’ OR ‘primary care’ OR ‘public health care’ OR ‘community care’)). Studies were included if they considered the role of occupational therapy with clients of all ages, with mental health issue, in a primary care context. The articles were screened by the first author and checked by the second author. The screening process is summarised in Figure 1.

## Results

A total of seventeen articles were included in the final review. A summary of the articles can be found in Table 1. Four common themes were identified across the included literature: 1. Variation in the definition of primary care used by occupational therapists; 2. Balancing generic and profession-specific roles; 3. Prioritization of physical health; and 4. Common interventions

**Table 1.** Articles included in review

Reference	Country	Number of participants	Type of study (time period)	Inclusion criteria and setting	Control/Intervention group	Findings
Cook, S. (2003). Generic and specialist interventions for people with severe mental health problems: Can interventions be categorized? British Journal of Occupational Therapy, 66(1), 17–24.https://doi.org/10.1177/030802260306600104	England	25	A case study of a primary care-based service. Involves retrospective analysis of routinely recorded case data for clients receiving interventions from occupational therapy staff(12 months)	Individuals with severe and enduring mental health problems that had lost contact with conventional community mental health services.Setting: General practice in inner-city area with high prevalence of severe mental illness	Intervention Group: 25 individuals with enduring psychotic conditions receiving 12 months of interventions from occupational therapy staff	1877 recorded interventionsInterventions were categorized into specific therapy (occupational therapy and psychological interventions) and generic care management using content analysis and categorical aggregation39.6% of interventions were categorized as care management, with 54.5% occupational therapy and 5.9% psychological interventions
Cook, S., & Howe, A. (2003). Engaging people with enduring psychotic conditions in primary mental health care and occupational therapy. British Journal of Occupational Therapy, 66(6), 236–246.https://doi.org/10.1177/030802260306600602	England	37 were identified for referral, 34 engaged with the service 28 received full 12 months intervention	Quasi-experimental, pre-test post-test design with a single cohort of GP patients(12 months)	Individuals with severe and enduring mental health problems that had lost contact with conventional community mental health services.Setting: General practice in inner-city area with high prevalence of severe mental illness	Intervention Group: 28 patients received 12 months of interventions (extended GP roles, occupational therapy, care management, liaison psychiatry).	Clinical and Social Outcomes: Significant improvements in social functioning (Social Functioning Scale), clinical symptoms (KGV Symptom Scale), and overall health (HoNOS) after 12 months.Cost Consequences: Favourable NHS costs compared to similar services.
Donnelly, C., Leclair, L., Hand, C., Wener, P., & Letts, L. (2023). Occupational Therapy Services in Primary Care: A scoping review. *Primary Health Care Research & Development*, *24*. https://doi.org/10.1017/s1463423622000123			Scoping review	Studies on occupational therapy roles in primary care for clients of all ages and conditions.Must involve an occupational therapist in a primary care setting.Excluded if not in English or French.		129 articles included.Identified 268 assessments and 868 interventions.Key interventions: community service navigation, chronic disease management, self-management education, health promotion, and falls prevention.Focus on adults and older adults.
Gallagher, A. M., Lyons, B., Houston, C., & Cummins, M. (2016). Exploring the current and potential role for occupational therapists in managing depression in primary care settings: Perspectives on occupational therapists in Ireland. *The Irish Journal of Occupational Therapy*, *44*(2).	Ireland	39 primary care occupational therapists, 10 community mental health occupational therapists	Mixed methods: quantitative cross-sectional survey and qualitative semi-structured interviews.	Primary Care Occupational Therapists (PCOTs): Currently employed in primary care for at least one year.Community Mental Health Occupational Therapists (CMHOTs): Currently working in community mental health with at least one year of postgraduate experience.Irish primary care settings		PCOTs:Low confidence in assessing (33.4%) and intervening (17.9%) for depression.Major barriers: prioritization of physical health, lack of time, training, and guidelines.Interest in addressing depression if given support and training.CMHOTs:Emphasized the need for occupational and functional assessments.Suggested interventions: skill teaching, time management, depression education, problem-solving, and vocational interventions.Identified barriers: focus on physical health, large caseloads, limited mental health training.
Trentham, B., Cockburn, L., & Shin, J. (2007). Health Promotion and Community Development: An application of occupational therapy in primary health care. *Canadian Journal of Community Mental Health*, *26*(2), 53–70. https://doi.org/10.7870/cjcmh-2007-0028	Canada		Case study	Occupational therapists working in primary health care settings using community development strategiesCommunity health centre in downtown Toronto		2 Group programmes:Community Action Group: Addressed issues like isolation and physical accessibility for older adults, leading to various community improvements (eg, increased sidewalk curb cuts, street benches).Getting On With Life and Its Challenges: A support and skill-building programme for adults with mental health challenges, fostering resilience through workshops and community activities.
Daaleman, C. E., Wright, S. T., & Daaleman, T. P. (2021). The effectiveness of occupational therapy for Mental Health Disorders in Primary Care: A Systematic Review. *British Journal of Occupational Therapy*, *85*(4), 224–230. https://doi.org/10.1177/03080226211058362	Included studies in Sweden, US, UK, and Ireland		Systematic review	An intervention that involved an occupational therapist in design and/or implementation.A quantitative outcome assessing symptoms related to a mental health disorder.A comparison group.Primary care or community dwelling patients and/or primary care clinical settings.		No significant differences in depression symptoms were found between the intervention and control groups at different time intervals. Some trials used interprofessional teams, including OTs, while others only included OTs. Anxiety symptoms showed no significant difference in one study, but another reported a reduction. The risk of bias was low in three studies and unclear in two.Limited evidence shows the impact of OT interventions on mental health outcomes in primary care. Due to varied interventions and inconsistent findings, further trials are needed.
Bolt, M., Ikking, T., Baaijen, R., & Saenger, S. (2019b). Scoping review: occupational therapy interventions in primary care. *Primary Health Care Research & Development*, *20*, e28, Article e28. https://doi.org/10.1017/S146342361800049X	Multiple European Countries		Position Paper	Occupational therapy interventions in primary care across Europe	Intervention group: Various examples of occupational therapy interventions identified through literature search and expert opinions	Recommendations:• Focus on occupational therapy services and professional development.• Make alliances.• Enhance public relations.• Improve financing and accessibility.
Eichler, J., & Royeen, L. (2016). Occupational therapy in the Primary Health Care Clinic: Experiences of two clinicians. Families, Systems, & Health, 34(3), 289–291. https://doi.org/10.1037/fsh0000226	US		Commentary – describes a pilot programme that implemented occupational therapy services	geriatric primary care setting and a university student health primary care clinic	Intervention group: elderly patients receiving OT services once a week for four hours initially, later expanded to twice weekly for four hours.University setting -college students receiving OT services addressing both medical and mental health needs.	Geriatric Primary Care Setting:Safe home recommendations, cognitive screenings, fall prevention, ADL and IADL modifications, home exercise, energy conservation, and work simplification.Anecdotal evidence of fewer office visits and rapid resolution of patient challenges.University Student Health Primary Care Setting:Habit and routine development, use of COPM, myofascial release, anxiety management strategies Evidence of reduced clinic visits and successful symptom resolution post-OT evaluation and intervention.
Scanlan, J. N., & Hazelton, T. (2019). Relationships between job satisfaction, Burnout, professional identity and meaningfulness of work activities for occupational therapists working in Mental Health. *Australian Occupational Therapy Journal*, *66*(5), 581–590. https://doi.org/10.1111/1440-1630.12596	Australia	118	Cross-sectional survey study	Must be an occupational therapist.Must be working in Australia.Must be working in mental health.Must have direct client contact as part of their position.Setting: Various mental health practice settings across Australia, including public health services, non-government services, private health services, and private practice.	Intervention group: occupational therapists working in mental health settings in Australia	study found that higher levels of meaningfulness in work activities were linked to greater job satisfaction, lower burnout, and stronger professional identity among occupational therapists in mental health. The “value to self” element was particularly influential. Additionally, no significant differences in work-wellbeing were observed between therapists in different settings or roles, though there was a trend towards higher job satisfaction in occupational therapy-specific roles.
Harrison, D. (2003). The case for generic working in Mental Health Occupational therapy. *British Journal of Occupational Therapy*, *66*(3), 110–112. https://doi.org/10.1177/030802260306600304	England		Opinion piece			
Henderson, P., Batten, R., & Richmond, J. (2015). Perceptions of the role of occupational therapy in Community child and adolescent mental health services. *Occupational Therapy in Mental Health*, *31*(2), 155–167. https://doi.org/10.1080/0164212x.2015.1035475	Australia	8	Qualitative Study	Non-occupational therapists with more than six months clinical experience. Currently working in community Child and Adolescent Mental Health Services (CAMHS) in Perth, WA	Intervention group: Participants from social work, psychology, psychiatry, and nursing disciplines	Limited Understanding: The study found a limited understanding of occupational therapy among community CAMHS team members.Team Membership: Occupational therapist’s ability to perform generic tasks was important for fitting into the multidisciplinary team.Perception Influenced by Exposure: Participants’ perceptions of occupational therapy were influenced by their past or current exposure to occupational therapists.Valued Perspectives: Different professional perspectives within the team, including occupational therapists, were valued.
Chamberlain, E., Truman, J., Scallan, S., Pike, A., & Lyon-Maris, J. (2019). Occupational therapy in primary care: Exploring the role of occupational therapy from a primary care perspective. *British Journal of General Practice*, *69*(688), 575–576. ×https://doi.org/10.3399/bjgp19×706517	England	20 participants3 occupational therapy students9 occupational therapy supervisors5 GPs3 wider primary care team members	Qualitative study(10 weeks)	Participants: Occupational therapy students and professionals currently working or placed in primary care settings in Hampshire, England.Settings: Four primary care settings including GP surgeries, a multidisciplinary nursing home team, and a community team.		Added Value of Occupational Therapy: Occupational therapists provided holistic care, addressing physical, psychological, and social needs.Support for Mental Health: Occupational therapists assisted patients with mental health issues who didn’t qualify for Community Mental Health Teams.Reduced Pressure on GPs: Occupational therapists alleviated GP workloads by addressing complex social and health issues.Development of Professional Identity: Students gained a stronger professional identity and better understanding of primary care.
Lambert, R. A. (2012). Routine general practice care for panic disorder within the lifestyle approach to managing panic study. *Mental Illness*, *4*(2), 91–95. https://doi.org/10.4081/mi.2012.e18	England	199 referrals received.117 patients were entered into the study.67 patients completed to final analysis	Randomized controlled trial(10 months)	Participants were aged between 16 and 65 years.Participants met DSM-IV criteria for panic disorder PD with/without agoraphobia.Stable medication dosage was required for at least 4 weeks before entry into the study.Participants were recruited from 15 GP practices in the East of England between 2001 and 2003.	Control:Unrestricted routine GP care (practice appointments, referrals, and prescriptions). (36 participants)Intervention group: Occupational therapy-led lifestyle treatment comprising lifestyle review of fluid intake, diet pattern, exercise, caffeine, alcohol, and nicotine. (31 participants)	Primary Outcome Measure: Beck Anxiety Inventory.Significant short-term clinical benefit for the lifestyle intervention, with it being at least as cost-effective as routine GP care over 10 months.
Harrison, D. (2005). Context of change in community mental health occupational therapy: Part one. *International Journal of Therapy and Rehabilitation, 12*(9), 396–399. https://doi.org/10.12968/ijtr.2005.12.9.19744	England	1 occupational therapist	Focused ethnography(observation was conducted over two periods of 3 days each, with a 2-week gap in between for preliminary analysis)	Setting: Community mental health team (CMHT) in the UK focused on services for older populations experiencing mental illness.The study involved observing one occupational therapist who worked in a CMHT.		Organizational Change: The study highlighted the impact of organizational changes on the occupational therapist’s role, including the shift to locality management and the loss of professional management structures.Generic vs. Profession-Specific Working: The occupational therapist balanced using generic skills (like depression assessments) with profession-specific skills (like traditional occupational therapy activities).Professional Identity: There was a tension between maintaining the unique focus of occupational therapy and integrating into a more generic mental health worker role.Uncertainty: The ongoing organizational changes created uncertainty and stress for the occupational therapist.
Harrison, D. (2005). Context of change in community mental health occupational therapy: Part two. International Journal of Therapy and Rehabilitation, 12(10), 444–447. https://doi.org/10.12968/ijtr.2005.12.10.24485	England	1 occupational therapist	Focused ethnography(observation was conducted over two periods of 3 days each, with a 2-week gap in between for preliminary analysis)	Setting: Community mental health team (CMHT) in the UK focused on services for older populations experiencing mental illness.The study involved observing one occupational therapist who worked in a CMHT.		
Muir, S., Henderson-Kalb, J., Eichler, J., Serfas, K., & Jennison, C. (2014). Occupational therapy in primary care: An emerging area of practice. In *OT Practice* (Vol. 19, Issue 15, pp. CE1–CE8). American Occupational Therapy Association, Inc.	US		Review and discussion of emerging practice models	Primary care settings in the United States		Identifies opportunities for integrating occupational therapy into primary care settings. Discusses new models of primary careHighlights the distinct role of occupational therapy in addressing physical, mental health, and paediatric care within primary care.Addresses the challenges of securing payment and documentation for occupational therapy services in primary care.
Cook, S., Chambers, E., & Coleman, J. H. (2009). Occupational therapy for people with psychotic conditions in community settings: a pilot randomized controlled trial. Clinical Rehabilitation, 23(1), 40–52. https://doi.org/10.1177/0269215508098898	England	44	Pilot randomized control trial(12 months intervention, with assessments at baseline, 6, 9, and 12 months)	Adults with schizophrenia or other psychotic conditions and functional problems; setting: two community mental health teams in a UK city	Control:Treatment as usual provided by non-occupational therapist multidisciplinary members of community mental health teams12 months of individualized occupational therapy in community settings as an adjunct to usual care	Both groups showed significant improvement on Social Functioning Scale and Scale for the Assessment of Negative Symptoms over 12 months.The occupational therapy group showed clinically significant improvements in four subscales of the Social Functioning Scale: relationships, independence performance, independence competence, and recreation. The treatment as usual group did not show clinically significant changes.The occupational therapy group showed a reduction in the number of participants with clinical levels of negative symptoms.

### Variation in definition of primary care used by occupational therapists

An exploration of available research indicated a discrepancy in how primary care was defined in relation to the role of occupational therapy. Two case study designs examining the categorization of generic and profession-specific interventions and the engagement of people with enduring psychotic conditions (respectively), used the terms ‘primary care’ and ‘community mental health care’ interchangeably (Cook, 2003; Cook and Howe, 2003).

More recent articles have echoed this difficulty with the definition and use of ‘primary care’. Donnelly *et al.* (2023) addressed this issue using a scoping review format to examine occupational therapy services in primary care. The article described the apparent discrepancy in how occupational therapy defines and understands primary care. Gallagher *et al.* (2016) was the only article that actively discriminated between primary care and community mental health care, examining the differences in roles and perceived barriers and enablers to occupational therapists in Irish care settings working with clients with a diagnosis of depression.

### Balancing generic and profession-specific roles

The interventions described across the included literature could be categorized as either a generic psychological or support role or an occupational therapy-specific role. This splitting of roles further blurs the perceived work of an occupational therapist in primary mental health care. The generic case management roles often offered to occupational therapists in primary care (Gallagher *et al.*, 2016), which may also be offered to health professionals with a background in various disciplines such as social work and psychology, may allow for a more flexible role for occupational therapists (Scanlan and Hazelton, 2019). The generic role may give room for substantial work within the multidisciplinary team, ensuring the integration of skills from other professions, such as cognitive behaviour therapy (Harrison, 2003; Henderson *et al.*, 2015). Cook and Howe (2003) distinctly differentiate between care management and occupational therapy, despite both roles being conducted by occupational therapists in the study. Cook (2003) explores the profession-specific or generic role debate in the United Kingdom, investigating the interventions undertaken by occupational therapists in a primary healthcare setting. It was found 40% of tasks fell into the category of generic care management. Cook *et al.* (2009) examine occupational therapy treatment in a similar setting, describing components of intervention as generic due to their requirement by all members of the multidisciplinary team. This highlights the necessary nature of some aspects of generic care management, such as risk management (Cook *et al.*, 2009). While older articles, Cook and Howe (2003), Cook (2003), and Cook *et al.* (2009), provide substantial insight into the two often competing aspects of primary care occupational therapy in mental health.

Interestingly, Gallagher *et al.* (2016) echoed Cook (2003) in emphasizing the pressure to work generically and the associated role blurring. Gallagher *et al.* (2016) found that community mental health occupational therapists view their role as more of a specialist than that of a primary care occupational therapist. Primary care was seen as a general area, with occupational therapists engaging in general care management. In contrast, community mental health and secondary care were seen as a specialist role, addressing complex presentations. Trentham *et al.* (2007) highlight the need for an occupational perspective to inform all aspects of work in occupational therapy, which is often lost when occupational therapists engage in generic roles in mental health.

In a two-part ethnographic study of an occupational therapist working in a newly established community mental health team, Harrison (2005a; 2005b) uncovered a strong belief that specialized training such as cognitive behavioural therapy gave occupational therapy the credibility that was felt to be much needed with a move away from occupational therapy specific assessments. Harrison (2005a; 2005b) emphasizes the uncertainty of the role of occupational therapy in primary mental health care, with the use of generic roles to give validity to the work of occupational therapists in this setting.

### Prioritization of physical health

Much of the literature described difficulty working with clients with mental health concerns, with primary care not seen as a practice area suited to working solely in mental health. Mental illness is rarely the sole health concern in a person’s life (Bolt *et al.*, 2019b), with physical illness highly prevalent in primary care spaces (Salisbury, 2012). This is exemplified in Bolt *et al.* (2019b)’s description of interventions undertaken when working with individuals with mental health concerns and chronic pain. Chamberlain *et al.* (2019) described the benefits of the holistic approach often favoured in primary care settings as allowing for an exploration of all aspects of clients while reducing pressure on general practitioner services. The lifestyle intervention practised with individuals with panic disorder is an example of utilizing this holistic approach, focusing on the individual’s fluid intake, diet pattern, exercise, and intake of caffeine, alcohol, and nicotine (Lambert, 2012).

In their review and discussion of emerging practice models in the United States, Muir *et al.*, (2014) sees primary care as an emerging model of practice fit to address both physical and mental health needs, explaining the role for both as enabling engagement in daily activities. However, Gallagher *et al.* (2016) emphasize the difficulties in integrating an occupational therapy practice that focuses on and makes little distinction between physical and mental health. The article, set in an Irish primary care practice context, uncovered issues in prioritizing physical health rather than mental health, with mental health issues seen as more time-consuming and less of a focus of their practice. Despite 90% of primary care occupational therapists interviewed in the study showing strong interest in addressing depression, confidence in assessing and providing intervention to those with depression was low (33.4% and 17.9%, respectively).

### Common interventions

Many articles discussed common interventions used by occupational therapists working with individuals with mental health concerns; however, these were often either guided by specific diagnoses (Gallagher *et al.*, 2016; Cook, 2003; Cook and Howe, 2003; Cook *et al.*, 2009) or were broad and not explicitly focused on primary healthcare (Trentham *et al.*, 2007; Daaleman *et al.*, 2021). Gallagher *et al.* (2016) and Eichler and Royeen (2016) discussed the use of formal information-gathering tools and assessments, such as the Model of Human Occupation and the Canadian Occupational Performance Measure (COPM). These assessment tools, as well as informal information-gathering processes, are used by occupational therapists to evaluate an individual’s environment, function, and skills. Daaleman *et al.* (2021) used a systematic review to examine interventions used by occupational therapists in primary care settings. Five studies were examined, which used various interventions, including work-directed rehabilitation through activity analysis, individual counselling to ensure readiness for work, physical activity, diet recommendations, goal setting, and psychoeducation. Similarly, Bolt *et al.* (2019b) identified supported employment as a crucial role of an occupational therapist in primary care. Focussing specifically on mental health, the interventions used included psychoeducation, motivational interviewing, and assertiveness through graded activity training.

While Eichler and Royeen (2016) is a commentary article, it provides interesting information on an informal case study in which a client with heart-related symptoms repeatedly ruled out as a physical condition was referred to an occupational therapist for probable anxiety. The occupational therapist used the COPM to assess his level of function and listened as the client spoke of his difficulties and dissatisfaction with his leisure skills. After developing goals with the occupational therapist, the client and the occupational therapist created a plan for de-escalating anxiety. The occupational therapist worked with him to identify several strategies that could be implemented within his current life and environment and discussed situations where he may apply the strategies. Despite the lower level of evidence, the article provides the most detailed description of a mental health intervention used by an occupational therapist in primary care. Even when compiled with other evidence from this literature review, a solid, well-rounded description of the role of an occupational therapist working with clients with mental health issues is still lacking.

## Discussion

While definitions of primary care and primary healthcare exist and are used in a general setting (World Health Organization, 2022), a definition of primary care in an occupational therapy setting has not been discovered in this literature review. Without this comprehensive, widely used, and accepted definition, difficulties in understanding the practice setting of articles lead to further issues in exploring the role of occupational therapists. To help readers understand a study’s findings and relevance to real-world practices, researchers must offer clear descriptions of the primary care setting and provide context about the healthcare models or services being studied. Without this, issues in understanding the role of occupational therapy in these settings and difficulties in researching these settings will remain.

The use of both generic and profession-specific roles in occupational therapy in primary mental health care leads to a further blurring of the role of occupational therapy in primary mental health care. The lack of occupational focus often seen with generic case manager roles creates isolation from the identity of occupational therapy (Ceramidas, 2010). It adds to the difficulty in understanding the role of occupational therapists in this setting.

While a holistic approach is a core value of occupational therapy, it is evident that occupational therapists in primary care lack confidence in working with people with mental health concerns. When an intervention is used in mental health, it often falls into a generic lifestyle approach, focusing on diet and exercise, without examining root causes or deeper issues relating to a person’s mental health. This approach when working with people with mental health concerns and the hesitancy in working with these communities often leads to a prioritization of physical illness creates further barriers to a cohesive definition of the role of occupational therapists in primary mental health care settings.

## Limitations

This report was based on a descriptive summary of the literature and therefore did not include quality appraisal or risk of bias check.

## Conclusion

Primary care settings exemplify ideals of equity, accessibility, and holism, which align with the professional values of occupational therapy. Occupational therapists are well-equipped to work with people with mental health concerns to support their participation in everyday activities that bring them meaning and purpose. This literature review illustrates a clear gap in the understanding of the role of occupational therapists working with people with mental illness in primary care settings, exacerbated by role blurring due to occupational therapists undertaking generic support and case management roles, and the prioritization of physical health in primary care. Further research is indicated which describes how occupational therapy may be most effectively utilized within a primary care setting and which examines any system or structural boundaries to integrating the role.

## References

[ref2] Arksey H and O’Malley L (2005) Scoping studies: towards a methodological framework. International Journal of Social Research Methodology 8, 19–32.

[ref38] Australian Institute of Health and Welfare (2025) *General Practice, Allied Health and Other Primary Care Services*. https://www.aihw.gov.au/reports/primary-health-care/general-practice-allied-health-primary-care

[ref5] Bolt M , Ikking T , Baaijen R and Saenger S (2019a) Occupational therapy and primary care. Primary Health Care Research & Development 20, e27. 32799974 10.1017/S1463423618000452 PMC6476805

[ref6] Bolt M , Ikking T , Baaijen R and Saenger S (2019b) Scoping review: occupational therapy interventions in primary care. Primary Health Care Research & Development 20, e28. 32799994 10.1017/S146342361800049X PMC6476367

[ref4] Burson K , Fette C and Kannenberg K (2017) Mental health promotion, prevention, and intervention in occupational therapy practice. The American Journal of Occupational Therapy 71, 7112410035p1–7112410035p19. 10.5014/ajot.2017.716S03 29309004

[ref7] Ceramidas DM (2010) A case against generalisation of mental health occupational therapy in Australia. Australian Occupational Therapy Journal 57, 409–416. 21091707 10.1111/j.1440-1630.2010.00876.x

[ref8] Chamberlain E , Truman J , Scallan S , Pike A and Lyon-Maris J (2019) Occupational therapy in primary care: exploring the role of occupational therapy from a primary care perspective. British Journal of General Practice 69, 575–576. 10.3399/bjgp19X706517 PMC6808580 31672831

[ref9] Cook S (2003) Generic and specialist interventions for people with severe mental health problems: can interventions be categorised? British Journal of Occupational Therapy 66, 17–24.

[ref10] Cook S , Chambers E and Coleman JH (2009) Occupational therapy for people with psychotic conditions in community settings: a pilot randomized controlled trial. Clinical Rehabilitation 23, 40–52. 19114436 10.1177/0269215508098898

[ref11] Cook S and Howe A (2003) Engaging people with enduring psychotic conditions in primary mental health care and occupational therapy. British Journal of Occupational Therapy 66, 236–246.

[ref12] Covidence (n.d) Covidence Systematic Review Software. Melbourne, Australia: Veritas Health Innovation.

[ref13] Daaleman CE , Wright ST and Daaleman TP (2021) The effectiveness of occupational therapy for mental health disorders in primary care: A systematic review. British Journal of Occupational Therapy 85, 224–230.

[ref14] Department of Health and Aged Care (2012) Mental Health Statement of Rights and Responsibilities: Glossary. Canberra, Australia: Department of Health and Aged Care.

[ref15] Donnelly C , Leclair L , Hand C , Wener P and Letts L (2023) Occupational therapy services in primary care: a scoping review. Primary Health Care Research & Development 24, e7. 36617849 10.1017/S1463423622000123 PMC9884533

[ref16] Ee C , Lake J , Firth J , Hargraves F , de Manincor M , Meade T , Marx W and Sarris J (2020) An integrative collaborative care model for people with mental illness and physical comorbidities. International Journal of Mental Health Systems 14, 83. 33292354 10.1186/s13033-020-00410-6 PMC7659089

[ref17] Eichler J and Royeen L (2016) Occupational therapy in the primary health care clinic: experiences of two clinicians. Families, Systems, & Health 34, 289–291. 10.1037/fsh0000226 27632545

[ref19] Gallagher AM , Lyons B , Houston C and Cummins M (2016) Exploring the current and potential role for occupational therapists in managing depression in primary care settings: perspectives on occupational therapists in Ireland. The Irish Journal of Occupational Therapy 44, 10–18.

[ref20] Harrison D (2003) The case for generic working in mental health occupational therapy. British Journal of Occupational Therapy 66, 110–112.

[ref21] Harrison D (2005a) Context of change in community mental health occupational therapy: part one. International Journal of Therapy and Rehabilitation 12, 396–399.

[ref22] Harrison D (2005b) Context of change in community mental health occupational therapy: part two. International Journal of Therapy and Rehabilitation 12, 444–447.

[ref23] Henderson P , Batten R and Richmond J (2015) Perceptions of the role of occupational therapy in community child and adolescent mental health services. Occupational Therapy in Mental Health 31, 155–167.

[ref24] Lambert RA (2012) Routine general practice care for panic disorder within the lifestyle approach to managing panic study. Mental Illness 4, 91–95. 10.4081/mi.2012.e18 PMC4253376 25478119

[ref25] Levac D , Colquhoun H and O’Brien KK (2010) Scoping studies: advancing the methodology. Implementation Science 5, 1–9. 20854677 10.1186/1748-5908-5-69 PMC2954944

[ref26] Muir S (2012) Occupational therapy in primary health care: we should be there. The American Journal of Occupational Therapy 66, 506–510. 22917116 10.5014/ajot.2012.665001

[ref27] Muir S , Henderson-Kalb J , Eichler J , Serfas K and Jennison C (2014) Occupational therapy in primary care: an emerging area of practice. OT Practice 19, CE1–CE8.

[ref28] Murtagh S , McCombe G , Broughan J , Carroll Á , Casey M , Harrold Á , Dennehy T , Fawsitt R and Cullen W (2021) Integrating primary and secondary care to enhance chronic disease management: a scoping review. International Journal of Integrated Care 21, 4. 10.5334/ijic.5508 PMC7880002 33613136

[ref1] Occupational Therapy Australia (2023) About occupational therapy. *Occupational Therapy Australia*. Available at https://otaus.com.au/about/about-ot (accessed 25 March 2023).

[ref30] Parker H (2001) The role of occupational therapists in community mental health teams: generic or specialist? British Journal of Occupational Therapy 64, 609–611.

[ref31] Salisbury C (2012) Multimorbidity: redesigning health care for people who use it. The Lancet 380, 7–9. 10.1016/S0140-6736(12)60482-6 22579042

[ref32] Sammells E , Logan A and Sheppard L (2022) Participant outcomes and facilitator experiences following a community living skills program for adult mental health consumers. Community Mental Health Journal 59, 428–438. 36074286 10.1007/s10597-022-01020-x PMC9981707

[ref33] Scanlan JN and Hazelton T (2019) Relationships between job satisfaction, burnout, professional identity and meaningfulness of work activities for occupational therapists working in mental health. Australian Occupational Therapy Journal 66, 581–590. 31304598 10.1111/1440-1630.12596

[ref34] Trentham B , Cockburn L and Shin J (2007) Health promotion and community development: an application of occupational therapy in primary health care. Canadian Journal of Community Mental Health 26, 53–70.

[ref35] WFOT (2012) About Occupational Therapy. London: WFOT.

[ref39] Wilding C (2009) Defining occupational therapy. In Occupational Therapy and Physical Dysfunction: Enabling Occupation, 6th ed. / 1st ed. Elsevier Churchill Livingstone, pp. 1–45.

[ref36] World Health Organisation (2025) Mental Health. Geneva: WHO.

[ref37] World Health Organization (2024) *Primary Health Care*. https://www.who.int/news-room/fact-sheets/detail/primary-health-care

